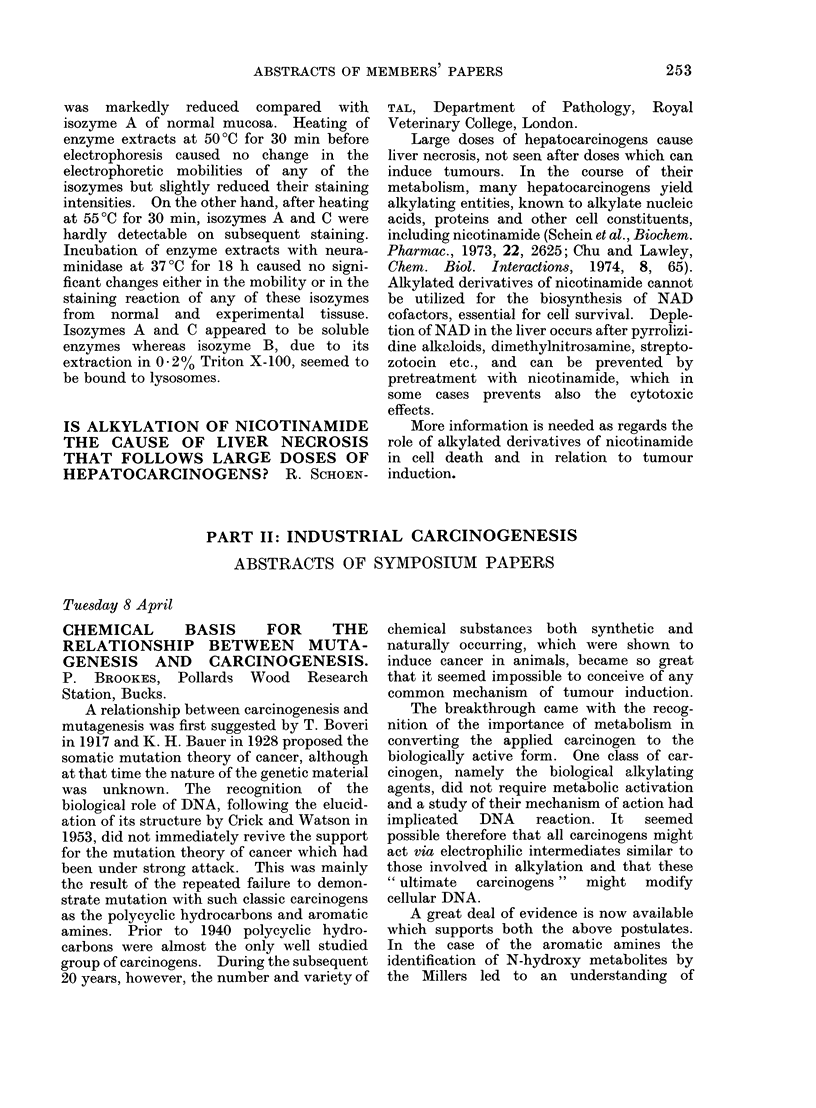# Proceedings: Is alkylation of nicotinamide the cause of liver necrosis that follows large doses of hepatocarcinogens?

**DOI:** 10.1038/bjc.1975.198

**Published:** 1975-08

**Authors:** R. Schoental


**IS ALKYLATION OF NICOTINAMIDE THE CAUSE OF LIVER NECROSIS THAT FOLLOWS LARGE DOSES OF HEPATOCARCINOGENS?** R. SCHOENTAL, Department of Pathology, Royal Veterinary College, London.

Large doses of hepatocarcinogens cause liver necrosis, not seen after doses which can induce tumours. In the course of their metabolism, many hepatocarcinogens yield alkylating entities, known to alkylate nucleic acids, proteins and other cell constituents, including nicotinamide (Schein *et al.*, *Biochem. Pharmac.*, 1973, **22**, 2625; Chu and Lawley, *Chem. Biol. Interactions*, 1974, **8**, 65). Alkylated derivatives of nicotinamide cannot be utilized for the biosynthesis of NAD cofactors, essential for cell survival. Depletion of NAD in the liver occurs after pyrrolizidine alkaloids, dimethylnitrosamine, streptozotocin etc., and can be prevented by pretreatment with nicotinamide, which in some cases prevents also the cytotoxic effects.

More information is needed as regards the role of alkylated derivatives of nicotinamide in cell death and in relation to tumour induction.